# Bruise dating using deep learning

**DOI:** 10.1111/1556-4029.14578

**Published:** 2020-09-29

**Authors:** Jhonatan Tirado, David Mauricio

**Affiliations:** ^1^ Department of Systems Engineering Universidad Nacional Mayor de San Marcos Lima Peru

**Keywords:** bruise dating, convolutional neural network, deep learning, MasNet

## Abstract

The bruise dating can have important medicolegal implications in family violence and violence against women cases. However, studies show that the medical specialist has 50% accuracy in classifying a bruise by age, mainly due to the variability of the images and the color of the bruise. This research proposes a model, based on deep convolutional neural networks, for bruise dating using only images, by age ranges, ranging from 0–2 days to 17–30 days, and images of healthy skin. A 2140 experimental bruise photograph dataset was constructed, for which a data capture protocol and a preprocessing procedure are proposed. Similarly, 20 classification models were trained with the Inception V3, Resnet50, MobileNet, and MnasNet architectures, where combinations of learning transfer, cross‐validation, and data augmentation were used. Numerical experiments show that classification models based on MnasNet have better results, reaching 97.00% precision and sensitivity, and 99.50% specificity, exceeding 40% precision reported in the literature. Also, it was observed that the precision of the model decreases with the age of the bruise.

## 1 INTRODUCTION

**FIGURE 1:**
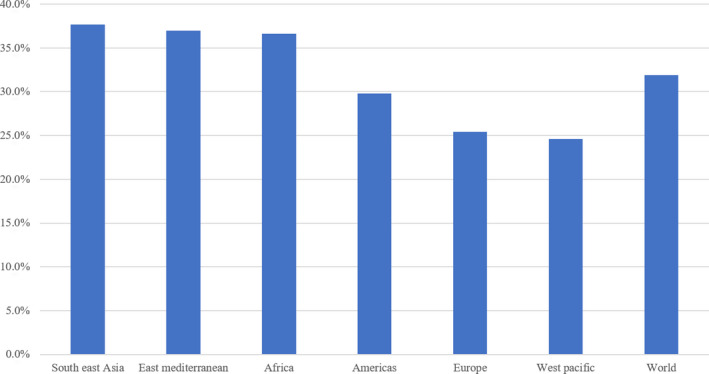
Bar graph showing rate of physical and/or sexual violence against women by region, according to the World Health Organization, as of 2013 [Color figure can be viewed at wileyonlinelibrary.com]

According to the latest report of the World Health Organization (WHO) [1], domestic violence against women is a global scourge, with a prevalence of physical or sexual abuse against women of just over 30% globally and 29.8% in Latin America (see Figure 1). Some victims of violence report these facts to the authorities, which require forensic clinical services such as bruise dating to support complaints, since the results of dating, used as evidence, can be decisive for justice. However, many of these complaints are filed due to lack of evidence or error in the dating of bruise, which aggravates the consequences of this scourge.

Bruise, also known as ecchymosis, is the internal bleeding of the skin due to the rupture of blood vessels, which is caused by an impact with an edgeless object, without tearing or cutting the skin. Therefore, blood escapes from blood vessels close to the surface of the skin being trapped under it [2]. This generates a coloration on the surface of the skin, which shows a series of colors that vary over time and can be visible for up to 30 days from its appearance, so the medical literature reports the use of color scales for dating of an bruise. This procedure consists in determining the age of this bruise; thus, the bruise is visualized *in situ* or through photographs, and the judgment of an expert is applied [3], who, in general, is a coroner, and its importance lies in being evidence fundamental in a trial for domestic violence or against women.

There are studies on the dating of bruise, from the medical and forensic point of view, where the use of histological analysis [4], genetic [5], chromatography [6], and visual inspection techniques are used, and also consider variables such as sex, age, and skin color of the person. However, due to the variability of the evolution of these bruises [2], there is still no reliable method to determine their age. This is mainly explained by biological variability, like location, size, depth, and degree of the injury, as well as race of the subject. Also, the biological status, such as diabetes, hemophilia, and leukemia, could affect the appearance and healing of bruises. Likewise, studies in the United States and Europe mainly include white‐skinned people in their experiments [7, 8, 9], which constitutes a different reality from other regions such as Latin America, where miscegenation is characteristic.

An exhaustive search in Web of Science, Scopus, and Google Scholar shows that to date there are no publications on bruise dating that use computer science techniques. However, there are artificial intelligence techniques that allow you to process images and differentiate for classification purposes. Publications show that the deep learning technique of artificial intelligence allows an accuracy of 90.16% for the diagnosis of glaucoma [10], 82.95% for psoriasis [11], and 82.3% for lung diseases [12]. In addition, in [13], it is applied to the diagnosis of melanoma, which affects the skin and presents variability in color, like bruise.

In this work, a deep learning model for bruise dating, based exclusively on images and convolutional neural networks, is proposed, for use on healthy living human beings only. MnasNet gave better results than the other three architectures evaluated for accuracy. In addition, it is optimized for use on mobile devices, so it must be small and fast, to allow a balance between accuracy and latency. To validate the model, the Tensorflow, Keras, and OpenCV libraries were used; then, tests were made with a dataset of 2140 images. Likewise, a protocol is proposed for capturing bruise photographs to guarantee image quality and high precision in the results.

This work is organized in five sections. In section two, a review of the literature on bruise dating is made. The bruise dating model and the photograph capture protocol are described in section three. The validation of the model, through the implementation of a system considering six classes by age ranges and the “Healthy skin” class, is presented in section four. Finally, the conclusions, limitations, and recommendations are presented in section five.

## 2 RELATED WORKS

There are few works on bruise dating, and these focus on the fields of medicine, biology, and genetics. In forensic medicine, for example, in [6], the use of tristimulus colorimetric is proposed as a method to objectively determine the color of an bruise in dark‐skinned people using the CIELAB color space, which reaches 95% accuracy of the color of bruise and that could be used for dating. In [15], tristimulus colorimetry is shown to be reliable for the evaluation of the color of a bruise generated experimentally with paintballs fired by compressed air guns. The use of a bilirubin meter is evaluated as a bruise dating method in [16], where it is found that the difference in bilirubin level between healthy skin and bruise has a peak between day 4 and 5, which decreases in the following days. An alternate light source, in the visible and ultraviolet spectrum, is used in [17] to evaluate its effectiveness in the detection of bruise, compared to white light. Detection is a previous step to bruise dating. In medicine and biology, the method of histological analysis is used for the dating of bruise; however, in [4], it is shown that it is not reliable due to the high variability of the response of human tissue to trauma due to stroke. In the field of genetics, in [5], the use of genetic expression signatures is proposed as a method to determine the strength of the impact and the age of a bruise in pigs, where differences of +/‐ two hours are obtained for ages ranging from one to 10 hours, and, due to its physiological and immunological similarity to human skin, it is suggested to extrapolate the results of the study to humans.

An exhaustive review to January 2020, in Web of Science, Scopus, and Google Scholar, based on the use of “bruise dating” search strings, shows that there are no bruise dating works through computational techniques such as artificial intelligence and image processing. However, there are works of image processing and artificial intelligence that have been developed for approximate problems to the dating of bruise. The “Relative Attribute SVM + Learning” algorithm is proposed in [18] for the estimation of age based on photographs of human faces; thus, it is considered that the presence of certain facial attributes at different ages keeps a relative order between age‐groups. In [19], it is sought to determine the age and gender of a person, and for this, panoramic dental X‐ray images are analyzed using image processing and a multilayer perceptron neural network. In another case, [20] proposes a deep hybrid model for classification by age range for human face images, where deep convolutional neural networks are used. The use of a Deep Belief Network, based on rough set theory for the classification of medical images of lung scans, is proposed in [12]. A new algorithm called “Ensemble Margin Instance Selection” (EMIS), based on Random Forest, is proposed in [21], to select the most informative data to optimize the classification of white blood cells. Finally, [22] proposes the use of a convolutional neural network to detect the gender (male or female) of a person based on a photograph of their eyes taken with the front camera of a smartphone, in everyday conditions with a normal camera. For the above, image processing and artificial intelligence could be used for bruise dating.

To obtain better results, the image preprocessing process, which involves the segmentation of the area of interest, is included in the image processing methods. [23] proposes using a deep convolutional neural network (DCNN) for the separation of the front and the bottom of an image, with a mean square error of 3.53%. [10] proposes an approach to the automatic diagnosis of glaucoma called “Super pixels for semi‐supervised segmentation” (SP3S) using segmentation, with an F‐score of 86.43%. [11] uses a deep convolutional neural network for the segmentation of skin psoriasis biopsy images, differentiating the dermis, epidermis, and non‐tissue regions, where 89% accuracy is achieved.

In relation to the bruise dating, the medical literature reports the use of temporal scales based on the coloring of the bruise to estimate its age. One of the pioneering scales is that of camps, which establishes a scale of levels, where the color of the bruise is red immediately after being inflicted, then it becomes dark purple or black, it turns green between the fourth and fifth day, yellow between day seven to 10, and disappears after 14 or 15 days. From there, various color scales have been established for bruise dating. A literature review on bruise dating scales until 1991 is performed in [2]. Table 1 shows four color scales for bruise dating, widely used in the literature. The scales are similar in terms of the sequence of changes in the color of the bruise, but differ in the times of these changes, although they all end with the green, then yellow color.

**TABLE 1:** Bruise dating scales and coloration. Adapted: [2]

Source	Bruise color	Bruise age
Camps	Red	Immediately
	Dark purple/black	Shortly after
	Green	4 to 5 d
	Yellow	7 to 10 d
	Disappearance	14 to 15 d
Glaister	Violet	Immediately
	Blue	day 3
	Green	5 to 7 d
	Yellow	8 to 10 d
	Disappearance	13 to 18 d
Polson and Gee	Dark red /red and black	less than 24 h
	Greenish	day 7
	Yellowish	day 14
	Disappearance	up to 30 d
Smith and Fides	Red	Immediately
	Purple/black	Shortly after
	Green	4 to 5 d
	Yellow	7 to 10 d, but small and superficial on day 3
	Disappearance	14 to 15 d

## 3 BRUISE DATING MODEL

A bruise dating model using deep learning is proposed, which allows the age of a bruise to be determined based on a photographic image of it, in living human beings. Its purpose is to determine the age of a bruise in a more accurate, objective, and quicker way, compared to the dating of bruise made by a human specialist (coroner and dermatologist). The main components are the protocol for image capture, image preprocessing, and the trained classification model based on convolutional neural networks.

**FIGURE 2:**
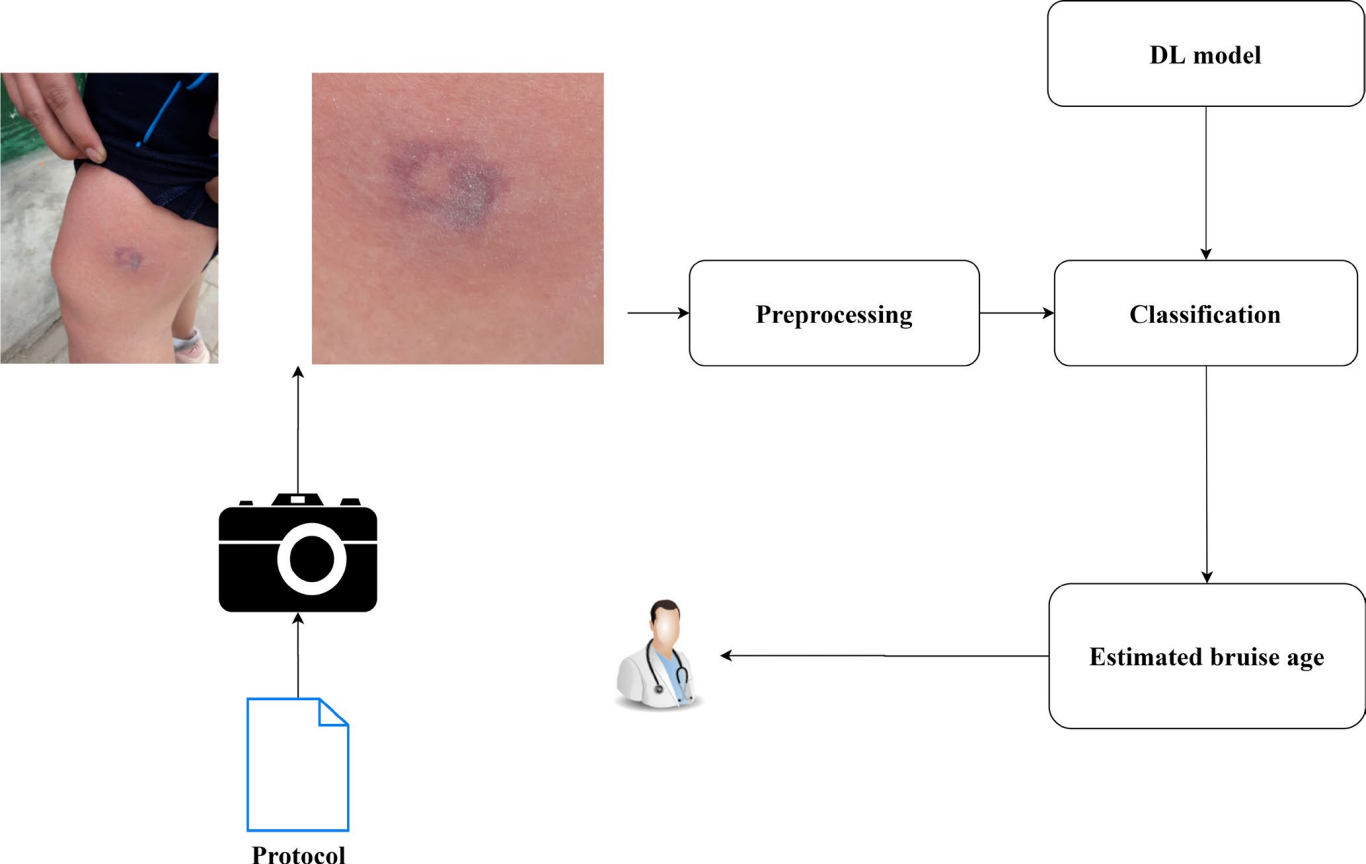
The Protocol icon represents the Bruise Image Capture Protocol document. The camera icon is the device used to obtain the bruise image. The Preprocessing, DL (deep learning) model, and Classification boxes represent the model components. The Estimated bruise age box represents the result reported by the model. The person icon represents the specialist who gets the result and uses it as needed [Color figure can be viewed at wileyonlinelibrary.com]

In Figure 2, the bruise dating model receives as input a photograph of a bruise that is obtained through a camera respecting a protocol. The image is then preprocessed to obtain a clean and segmented image of the bruise. This is then processed by the classifier that implements a previously trained convolutional network model, with which the estimated age of the bruise is determined, this being the result of the model.

The use of convolutional neural networks is justified because the dating of bruise has low accuracy rates, thus reaching 40% for bruises less than 48 hours, a percentage that decreases as the age of the bruise increases [7]. In addition, they present good results, comparable to medical specialists, for similar problems such as sarcoma [24] and melanoma [13] (they affect the skin and its diagnosis is visual based on images).

Therefore, the objective of this study is to build a bruise dating model that manages to exceed the accuracy reported in the literature.

### 3.1 Protocol for image capture

In order for the bruise dating model to estimate the correct age of the bruise, the photographs must be captured following a series of steps defined under specific conditions to guarantee the quality of the bruise photographs, which constitute the main and only characteristic used in this study. Table 2 describes the protocol for bruise image capture.

**TABLE 2:** Bruise image capture protocol

ID	Step
P01	Illuminate the environment properly, using either natural or white light
P02	Turn off the camera flash
P03	Set the camera at maximum resolution. The minimum resolution is 1024 × 768 pixels
P04	Ask the person to discover the skin in the injured area, so the skin is free of clothing, jewelry, accessories, or other objects
P05	Ask the person to clean the injured area, to remove substances such as sand, soil or blood
P06	Hold the camera with both hands perpendicular to the bruise, in vertical or horizontal mode
P07	Place the camera at a distance between 30 and 35 centimeters from the bruise
P08	Focus the image so that the bruise is in the center
P09	Verify that no shadows are cast on the area to be photographed
P10	Capture one photograph for each bruise injury. Verify the images are not blurred, shadowed, out of focus, too close or too far
P11	Verify that the photographs have been captured correctly and stored in the camera's memory
P12	Transfer the photographs from the camera to a folder on the computer where it will be processed by the bruise dating model
P13	Avoid reducing the size of the files or losing the resolution of the images during the transfer of the files

### 3.2 Data preprocessing

The input, both for the classification process and for the learning process, is the photographs of the bruise captured following the protocol and digitized in a repository. These images are preprocessed using the binarization of the grayscale image to segment the bruise, calculate the centroid of the bruise and trim the image to a size of 400 × 400 pixels.

The steps in this process are as follows:

1. Convert a copy of the original image to grayscale.
2. Binarize the grayscale image.
3. Calculate the position (X, Y) of the centroid of bruise, using the Moments function of the OpenCV library.
4. Trim a 400 × 400 pixels portion of the original color image, with its center located in the centroid of the bruise, calculated in step 3.
5. Save the image, obtained in step 4, as a new file.

**FIGURE 3:**
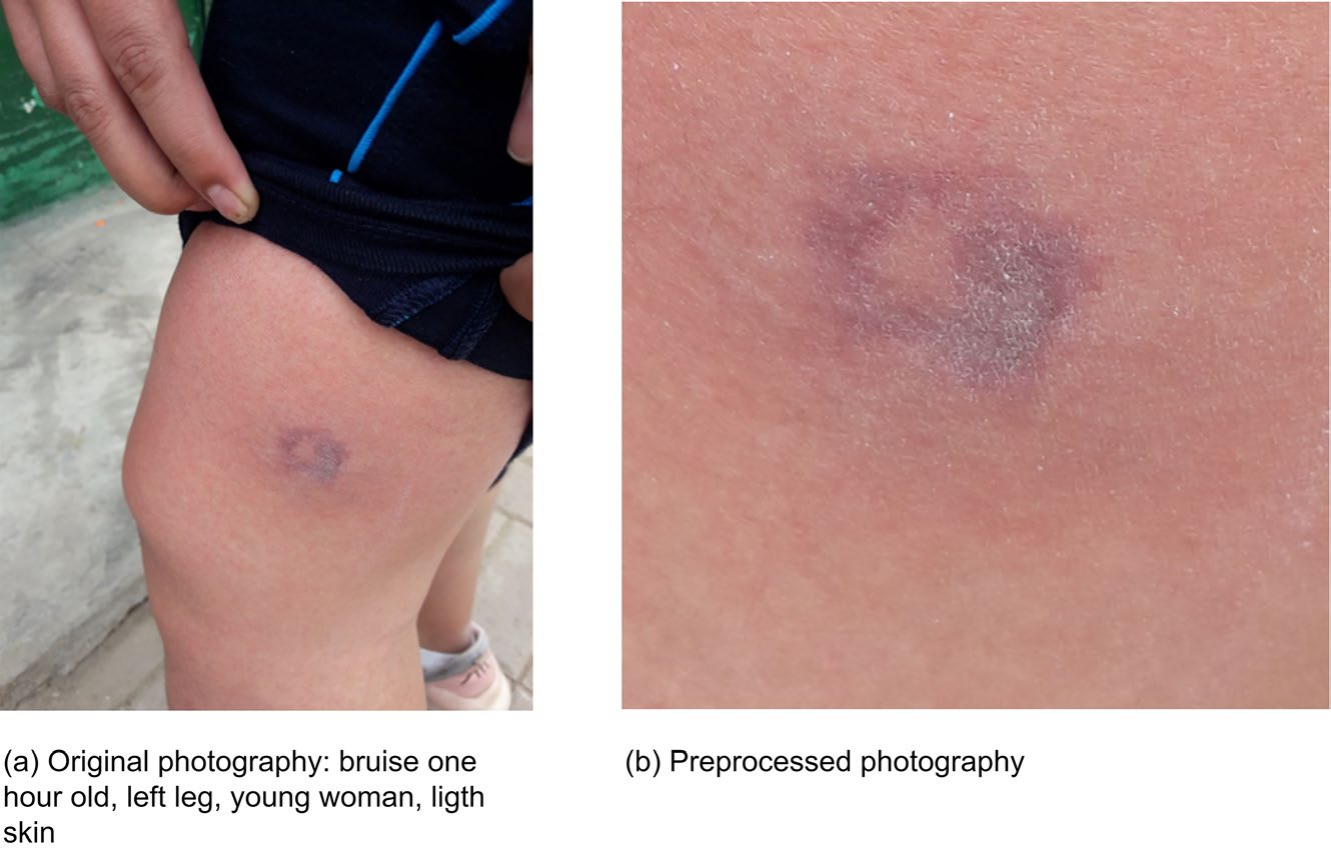
Raw, original bruise image, and preprocessed image of an experimental bruise [Color figure can be viewed at wileyonlinelibrary.com]

In this way, the original photographs are preprocessed, and a square‐shaped image is obtained with the bruise centered in it. To do this, a script was developed using the Python programming language and the OpenCV library. Figure 3 shows an original and preprocessed photograph, the latter of 400 × 400 pixels, with the centroid of the bruise in the center of the image.

### 3.3 Learning

The learning model for bruise classification by age range is based on convolutional neural networks. The input is the photograph, captured following the protocol, and the actual age of the injury. The photographs must be previously preprocessed and organized in folders, according to the age (in days) of the injury. In addition, the classes to be used must be established (see, e.g., the scales in Table 1), and the images must be grouped into folders according to these classes. In case the dataset is not balanced, it is suggested to use the data augmentation technique, which will allow greater precision [25].

For learning, it is suggested to evaluate some variants of convolutional neural networks, such as Inceptionv3 [26], Resnet50 [27], MobileNet [28], and MnasNet [14].

Previously, 10% of the images for each class should be set apart to be used as the "test" dataset, to prevent data leakage, and to avoid using test data during training.

**FIGURE 4:**
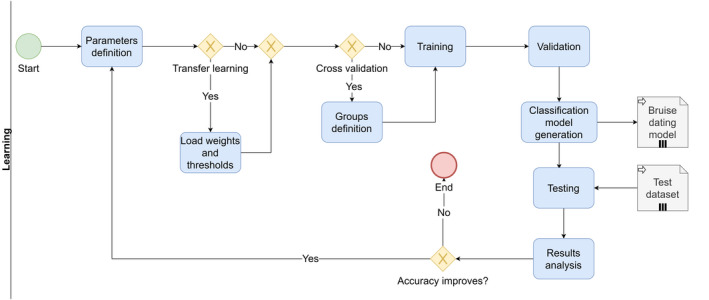
Process map (in BPMN notation) for the learning cycle of the artificial intelligence model. Green circle is the start, red circle is the end of the process. Arrows are transitions between tasks, represented by light blue boxes. Yellow diamonds represent a decision point. File icons represent documents or files. BPMN: business process management notation [Color figure can be viewed at wileyonlinelibrary.com]

In Figure 4, the experimentation cycle to find the best bruise dating model is shown. This cycle is repeated as many times as necessary, with variants such as the use or not of cross‐validation and transfer learning. Each cycle can include the execution of training, validation, testing, and analysis of results activities multiple times, until the model with greatest precision for bruise dating is obtained. All generated models are sent to a file server in the cloud. Finally, the bruise dating model with highest accuracy is selected.

### 3.4 Classification

The convolutional neural network model that obtained the highest precision during the learning phase can be used to classify new photographs of bruise. The model consists of a file that contains the structure, weights, thresholds, and parameters of the network. The classification model can be implemented as an API (Application Programming Interface), to be consumed by a mobile or web application, or embedded in an off‐line mobile application, for bruise classification.

The input of the classification model is a bruise photograph, and the output is a probability distribution that indicates the bruise belongs to one of the established classes.

## 4 VALIDATION

The validation process of this study consists in conducting numerical experiments. For this, four DCNN models were trained with the dataset detailed in section (dataset). Then, the learning models resulting from each neural network were evaluated using the metrics indicated in section (metrics).

**FIGURE 5:**
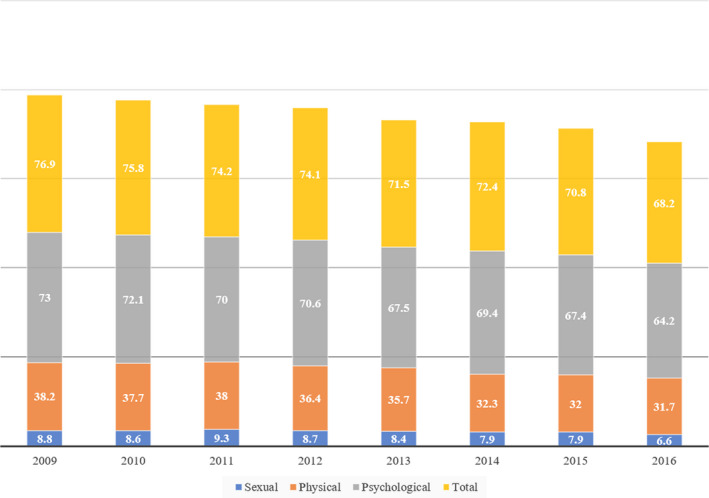
Bar graph showing the rate of violence against women exercised by her husband or partner in Peru. Sexual violence in blue, physical violence in orange, psychological violence in gray, and total violence in yellow color [Color figure can be viewed at wileyonlinelibrary.com]

The validation was applied to the Peruvian case, where the levels of violence against women (see Figure 5) reach 68.2%, and 31.7% for physical violence [29], a percentage slightly lower than the world average that reaches 31.9% (see Figure 1), and the majority of the population is mestizo, with a skin color that is not black or white. In addition, it should be considered that most research includes only white‐skinned people [2, 7, 8, 9, 15, 30, 31, 32], and there is a study that indicates that yellow coloration of a bruise is not visible in people with dark skin [6].

### 4.1 Dataset

This study requires the construction of a dataset big enough to train a neural network and classify bruises according to their age. For this, a controlled experiment was carried out using a bruise generation method, like the one used in [15] and [17]. Two paintball matches were held, with a difference of 30 days between them. The game consists of firing paintballs with compressed air guns. In this scenario, players often get bruises, despite safety measures such as helmets, vests, protectors, and power limits of weapons. In total, 11 volunteers (one participated in both matches) of mixed skin (four women and seven men), between 25 and 68 years, took five daily photographs of bruise following the data capture protocol detailed in section 3.1, at the same time of the day, from game day (day one) to the day 30. Only photographs of unprotected areas of the body (lower, upper limbs, and buttocks) were obtained, making a total of 18 different bruises. Table 3 shows the characteristics of the volunteers, including sex, age, skin color, health conditions, and location of the bruise or bruises. It also shows the camera used to obtain the images, the time of the day, and the location where the image was taken. Thus, the images are like a real and heterogeneous situation.

**TABLE 3:** Description of test subjects

ID	Sex	Age	Skin photo type (Fitzpatrick)	Bruise location	Camera model	Location	Time of the day
S1	Female	38	IV	Arm	Samsung J7	Home	Morning
				Buttock			
S2	Male	35	III	Arm	Samsung J7	Home Work	Morning
				Leg			
S3	Female	31	IV	Arm	Huawei Y6	Home	Night
S4	Male	68	IV	Arm	Samsung J7	Home	Night
				Back			
S5	Female	28	III	Leg	Samsung J1	Home	Afternoon
S6	Female	40	IV	Chest	Huawei P30	Home	Night
S7	Male	37	IV	Arm	Samsung A70	Work	Night
S8	Male	22	III	Arm	LG K50	Work	Afternoon
S9	Male	40	IV	Arm	Huawei P8	Home	Afternoon
				Leg			
S10	Male	22	IV	Arm	Huawei Y5	Work	Afternoon
S11	Male	24	IV	Arm	ZTE Blade A602	Home	Afternoon

This way, over a period of 60 days, bruise photographs were collected and a dataset was built with the characteristics detailed in Table 4, which includes the number of images that were used for training, validation, and testing of the model, for each of the classes used by forensic doctors in Peru [33]. The dataset is available on request.

**TABLE 4:** Dataset photographs distribution per class

Class	Training	Validation	Test	Total
Few hours to 2 d	179	23	22	224
3 d	85	11	11	107
4 to 6 d	250	31	31	312
7 to 12 d	450	56	56	562
13 to 17 d	200	25	25	250
More than 17 d	453	57	56	566
Healthy skin	95	12	12	119
Total	1712	215	213	2140

The total number of photographs estimated for the experiment is 5400; however, photographs of bruise on fingers are excluded, since the photographs contain more than one bruise. In addition, despite the established protocol, some participants did not submit the photographs daily, or the photographs presented shadows or blur, so a total of 2140 photographs were collected, of which 2021 contain a bruise, and 119 photographs show healthy skin. In addition, it has been observed that bruises in the two people with darkest skin were visible until the fifth day, while in the two people with lightest skin they were visible even until day 30.

### 4.2 Implementation

The Python programming language and TensorFlow, Keras, pandas, and numpy libraries were used to build, train, validate, and test the different versions of the learning models. The Inceptionv3, Resnet50, and MobileNet models were trained in a notebook and a virtual machine in the cloud, while the MnasNet model was trained using Google's AutoML Vision service.

As part of the training script of the bruise dating model, the preprocessed images were resized to 224 × 224 pixels.

For the transfer learning, the InceptionV3, Resnet50, and MobileNet models, included in the Keras library, pre‐trained in the ImageNet dataset, were used. No transfer learning was used for the training of the MnasNet model.

In the case of cross‐validation, the dataset was divided into 10 groups, which were used for training and validation. Previously 10% of the photographs by each class were separated for testing the models. In addition, the stochastic gradient descent algorithm was used for the optimization of Inceptionv3, Resnet50, and MobileNet models, with a learning rate of 0.0001, and batch size equal to of 32. The models were trained with 100, 200, and 1000 times. If the validation accuracy stopped improving for three consecutive epochs, the training was stopped, and another variant was tested.

In summary, 20 models of bruise dating were trained using four variants of neural networks, with or without cross‐validation, transfer learning, and different number of training epochs (see Table 5). The MnasNet model architecture and its parameters were determined by Google's AutoML Vision service, which makes an automated search for a neural network architecture optimized for mobile devices in terms of accuracy and latency [14]. A recurrent neural network (RNN) is used to generate the candidate architectures to be evaluated, then trains, tests the models, and feeds the RNN to generate an optimized architecture, repeating the cycle. The finally generated model consists of a DCNN trained in the specific dataset and optimized for execution in mobile devices. This model was embedded in an Android mobile application, for bruise dating in off‐line mode. The dataset, available on request, and access to the Google AutoML Vision service are enough to replicate the results of the best model.

**TABLE 5:** Trained bruise dating models

Id	Model	Cross‐validation	Transfer learning	Epochs	Classes	Data augmentation
M1	InceptionV3	No	No	100	7	No
M2	InceptionV3	No	No	200	7	No
M3	InceptionV3	No	No	1000	7	No
M4	InceptionV3	No	Yes	100	7	No
M5	InceptionV3	No	Yes	200	7	No
M6	InceptionV3	No	Yes	1000	7	No
M7	InceptionV3	Yes	No	100	7	No
M8	InceptionV3	Yes	No	200	7	No
M9	InceptionV3	Yes	No	1000	7	No
M10	InceptionV3	Yes	Yes	100	7	No
M11	InceptionV3	Yes	Yes	200	7	No
M12	InceptionV3	Yes	Yes	1000	7	No
M13	Resnet50	No	No	100	7	No
M14	Resnet50	No	Yes	100	7	No
M15	MobileNet	No	No	100	7	No
M16	MobileNet	No	Yes	100	7	No
M17	MnasNet^a^	‐‐‐	‐‐‐	‐‐‐	6^b^	No
M18	MnasNet^a^	‐‐‐	‐‐‐	‐‐‐	5^c^	No
M19	MnasNet^a^	‐‐‐	‐‐‐	‐‐‐	7	No
M20	MnasNet^a^	‐‐‐	‐‐‐	‐‐‐	7	Yes

^a^ The parameters were determined by Google's AutoML Vision service.

^b^ All classes except “Healthy skin.”

^c^ All classes except "More than 17 d" and “Healthy skin.”

The 16 variants of the trained models based on InceptionV3, Resnet50, and MobileNet differ in the use or not of cross‐validation, transfer learning, and number of training epochs. The models based on MnasNet, from M16 to M20, differ in the inclusion or not of the classes "More than 17 days," "Healthy skin," and the use or not of data augmentation. The data augmentation for M20 was obtained by duplicating the data of the “Healthy skin” class.

### 4.3 Metrics

The following metrics were used to evaluate and test the learning models:

- Precision (PRE). Rate of instances classified correctly.
- Sensitivity (SEN). True‐positive rate, that is, values classified as positive when they are positive. Correctly identify instances within a class.
- Specificity (SPE). True‐negative rate, that is, values classified as negative when they are negative. Correctly identify instances that do not belong to a class.

In this work, the goal is to obtain a model with high precision and sensitivity, since the most important thing is to classify a bruise correctly according to its age. Achieving high specificity is not a priority in this case, but it would be convenient to achieve a balance between sensitivity and specificity.

### 4.4 Results

Table 6 shows the precision, in training and validation, obtained by the 20 models indicated in Table 5.

**TABLE 6:** Precision (PRE) for each trained model

Model	Training Precision (%)	Validation Precision (%)
M1	39.88	41.35
M2	86.56	40.22
M3	33.97	33.85
M4	53.57	32.14
M5	81.31	46.43
M6	89.93	42.86
M7	46.10	32.96
M8	54.08	38.55
M9	54.91	31.28
M10	30.97	26.26
M11	37.77	18.75
M12	56.25	56.25
M13	30.66	17.32
M14	96.04	43.58
M15	89.71	38.55
M16	25.55	25.70
M17	97.41	97.41
M18	96.53	96.53
M19	97.13	97.13
M20	97.78	97.78

Overfitting was presented for the M1‐M16 models, which is explained because the validation accuracy is much lower than the training accuracy. On the other hand, the models that present greater precision in the validation are M17‐M20, based on the MnasNet model (see Table 5). These models were exposed to a more detailed analysis, as shown in Table 7, to determine the best one for bruise dating.

**TABLE 7:** Results for bruise dating models based on MnasNet

Class	M17			M18		
	PRE %	SEN %	SPE %	PRE %	SEN %	SPE %
Few hours to 2 d	95.00%	95.00%	100.00%	95.00%	95.00%	100.00%
3 d	100.00%	100.00%	100.00%	91.00%	91.00%	100.00%
4 to 6 d	97.00%	97.00%	99.20%	100.00%	100.00%	95.75%
7 to 12 d	100.00%	100.00%	98.00%	96.00%	96.00%	98.75%
13 to 17 d	96.00%	96.00%	99.00%	96.00%	96.00%	100.00%
More than 17 d	93.00%	93.00%	100.00%	‐‐‐	‐‐‐	‐‐‐
Healthy skin	‐‐‐	‐‐‐	‐‐‐	‐‐‐	‐‐‐	‐‐‐
Average	97.00%	97.00%	99.37%	96.00%	96.00%	98.90%

Class	M19			M20		
	PRE %	SEN %	SPE %	PRE %	SEN %	SPE %
Few hours to 2 d	100.00%	100.00%	99.33%	95.00%	95.00%	100.00%
3 d	100.00%	100.00%	100.00%	96.00%	96.00%	100.00%
4 to 6 d	100.00%	100.00%	99.33%	94.00%	94.00%	100.00%
7 to 12 d	96.00%	96.00%	100.00%	96.00%	96.00%	97.17%
13 to 17 d	96.00%	96.00%	99.17%	96.00%	96.00%	98.50%
More than 17 d	95.00%	95.00%	98.67%	95.00%	95.00%	99.00%
Healthy skin	92.00%	92.00%	100.00%	94.00%	94.00%	99.67%
Average	97.00%	97.00%	99.50%	95.00%	95.00%	99.19%

Table 7 shows that M19 has the highest average precision, sensitivity, and specificity, being the model chosen for bruise dating.

As can be seen in the Confusion Matrix (table 8), high precision was obtained for all classes, where the "Healthy skin" class is the least accurate with 92%, which is still high. For the “From 7 to 12 days” class, the model has a 4% error, predicting an age of “few hours to 2 days,” a class not adjacent to the real class. This may be due to an error following the image capture protocol. Something similar, although to a lesser extent, happens with the class "from 13 to 17 days."

**TABLE 8:** Confusion matrix for M19 (%)

Class	Predicted						
Real	Few hours to 2 d	3 d	4 to 6 d	7 to 12 d	13 to 17 d	More than 17 d	Healthy skin
Few hours to 2 d	100%	0%	0%	0%	0%	0%	0%
3 d	0%	100%	0%	0%	0%	0%	0%
4 to 6 d	0%	0%	100%	0%	0%	0%	0%
7 to 12 d	4%	0%	0%	96%	0%	0%	0%
13 to 17 d	0%	0%	4%	0%	96%	0%	0%
More than 17 d	0%	0%	0%	0%	5%	95%	0%
Healthy skin	0%	0%	0%	0%	0%	8%	92%

## 5 CONCLUSIONS

This work has introduced a model for bruise dating in living human beings, based on deep learning, which considers a protocol for obtaining images, a preprocessing procedure, and a classification model based on deep convolutional neural networks.

The numerical results on 20 configurations of classification models, tested on 213 images, show that models based on InceptionV3, Resnet50, and MobileNet have overfitting and dating precision less than 57%. Meanwhile, MnasNet‐based models achieve precision greater than 95%, the best results being 97% precision, 97% sensitivity, and 99.5% specificity, for the model with 7 classes and no data augmentation, a result far exceeding 40% reported in the literature to date. The results also show that the quality of precision decreases as the age of the bruise increases. In the best model, this is manifested from the seventh day. This can be explained because the visual information of the bruise is lost as time passes. These results confirm the proposed model is suitable for bruise dating, given that it has presented 97% precision for bruise dating on people of mestizo complexion, far above the 50% precision obtained by experts through images of white people, suggesting the model could be used for other skin colors.

A limitation of the current results is that they are based on images obtained in a controlled experiment and heterogeneous context, to guarantee the accuracy of the information, given the difficulty to get bruise images of cases of violence. A future work is extending the proposed model for some aspects of the physical violence, such as the used object, intensity, and geographical location of the event of violence.

The main limitation of this study and the proposed model is that it is based exclusively on images of healthy people for bruise dating. Thus, a future work is to extend the model to include the biological variability, such as age, sex, skin color, and race of the subject, but also location, size, depth, and degree of injury. Another future work is extending the model for various biological statuses (e.g., people with chronic diseases such as diabetes).
